# Increased glycolysis and cellular crosstalk in eosinophilic chronic rhinosinusitis with nasal polyps

**DOI:** 10.3389/fimmu.2024.1321560

**Published:** 2024-02-20

**Authors:** George X. Huang, Michael V. Mandanas, Sarah Djeddi, Daniela Fernandez-Salinas, Maria Gutierrez-Arcelus, Nora A. Barrett

**Affiliations:** ^1^ Division of Allergy and Clinical Immunology, Brigham and Women’s Hospital, Boston, MA, United States; ^2^ Department of Medicine, Harvard Medical School, Boston, MA, United States; ^3^ Division of Immunology, Boston Children’s Hospital, Boston, MA, United States; ^4^ Department of Pediatrics, Harvard Medical School, Boston, MA, United States; ^5^ Broad Institute of MIT and Harvard, Cambridge, MA, United States

**Keywords:** chronic rhinosinusitis, single cell RNA sequencing, epithelium, glycolysis, epithelial mesenchymal interaction

## Abstract

**Introduction:**

Chronic rhinosinusitis (CRS) is a chronic inflammatory disease of the sinonasal mucosa with distinct endotypes including type 2 (T2) high eosinophilic CRS with nasal polyps (eCRSwNP), T2 low non-eosinophilic CRS with nasal polyps (neCRSwNP), and CRS without nasal polyps (CRSsNP).

**Methods:**

Given the heterogeneity of disease, we hypothesized that assessment of single cell RNA sequencing (scRNA-seq) across this spectrum of disease would reveal connections between infiltrating and activated immune cells and the epithelial and stromal populations that reside in sinonasal tissue.

**Results:**

Here we find increased expression of genes encoding glycolytic enzymes in epithelial cells (EpCs), stromal cells, and memory T-cell subsets from patients with eCRSwNP, as compared to healthy controls. In basal EpCs, this is associated with a program of cell motility and Rho GTPase effector expression. Across both stromal and immune subsets, glycolytic programming was associated with extracellular matrix interactions, proteoglycan generation, and collagen formation. Furthermore, we report increased cell-cell interactions between EpCs and stromal/immune cells in eCRSwNP compared to healthy control tissue, and we nominate candidate receptor-ligand pairs that may drive tissue remodeling.

**Discussion:**

These findings support a role for glycolytic reprograming in T2-elicited tissue remodeling and implicate increased cellular crosstalk in eCRSwNP.

## 1 Introduction

In the past 5 years, single cell RNA sequencing (scRNA-seq) has allowed an unprecedented assessment of tissue immunology in human health and disease. This technology has allowed us to identify novel immune and stromal cell types, recognize unexpected heterogeneity in immunocyte subsets, and establish trajectories of cell differentiation in human tissues (1, 2). While much of this first wave of studies includes observations about ever smaller subsets of cells, as the technology and computational programs have advanced, and as datasets from larger numbers of patients have become available, we have the opportunity to look at higher level immune architecture in tissues (3, 4), so called systems immunology. This includes assessing shared programs across immune and stromal cells, identifying cell-cell interactions in health and disease, defining disease endotypes, and integrating transcriptional data with genetic, epigenetic, and protein data (5, 6).

Chronic rhinosinusitis (CRS) is a chronic inflammatory disorder of the sinonasal mucosa which is highly associated with asthma. Two main forms of CRS are chronic rhinosinusitis without nasal polyps (CRSsNP) and chronic rhinosinusitis with nasal polyps (CRSwNP) (7). In western countries, CRSwNP is largely characterized as a type 2 (T2) inflammatory disease with robust infiltration of eosinophils, mast cells, basophils, Th2A cells, and IgE and IgG4 producing plasma cells (7, 8). CRSwNP is also associated with significant defects in epithelial function, including the reduced expression of epithelial tight junction proteins, anti-oxidant enzymes, and innate immune molecules (9). It is also associated with the expansion of a basal epithelial cell (basal EpC) population which fails to differentiate but produces alarmins such as IL-33 and TSLP that promote T2 inflammation (10, 11). In Asia, CRSwNP includes both eosinophilic (eCRSwNP) and non-eosinophilic (neCRSwNP) subtypes (8, 12, 13). While eCRSwNP appears similar to that described in western countries, the neCRSwNP subtype has not been well characterized and is likely more heterogeneous, with some studies reporting T1-, T3-, and neutrophil-predominant endotypes (8, 14).

Given the heterogeneity of CRS subtypes we hypothesized that analysis of scRNA-seq datasets would reveal cell states and interactions that were specific to CRS subtypes. Thus, in this report we analyzed the Wang dataset (13), a large human scRNA-seq dataset which includes >70,000 sinonasal cells from a variety of disease types, including healthy controls (n=5), CRSsNP (n=5), neCRSwNP (n=5), and eCRSwNP (n=6). We specifically explored immunometabolism and cell-cell interactions through transcriptomic assessment to identify aberrant cell states and cellular crosstalk in CRSwNP.

## 2 Methods

Single cell RNA sequencing (scRNA-seq) FASTQ files of nasal mucosa from healthy controls (n=5), CRSsNP (n=5), neCRSwNP (n=5), and eCRSwNP (n=6) were downloaded from Genome Sequence Archive [HRA000772] (13) and processed as previously described (15). Clustering and sub-clustering reproduced the global structure reported by Wang et al. (13) with 47 major cell types (including 9 EpC cell types, 5 stromal cell types, and 33 immune cell types) (**Supplementary Figure 1A**).

Cell type quantification was performed within the chronic rhinosinusitis (CRS) conditions (CRSsNP, neCRSwNP, and eCRSwNP) due to differences in collection methods between healthy control and CRS tissue (13). Gene set module scores for various human MSigDB gene sets (16) - including metabolic gene sets such as Mootha gene sets (17), Gene Ontology Biological Processes (GOBP) gene sets (18), and Reactome gene sets (19, 20) - were calculated for each cell using the Seurat AddModuleScore function with default parameters (21, 22). For correlation between Mootha glycolysis scores and the GOBP module scores or Reactome module scores, any overlapping genes were removed from the GOBP or Reactome gene set prior to module score calculation. CellPhoneDB v2 (23) was used to nominate cell-cell ligand-receptor interactions within each sample, using a expression threshold of 0.3. Interactions with p-value less than 0.05 were considered significant.

Statistical comparisons were performed using non-parametric Wilcoxon tests and non-parametric Spearman correlation tests. Where applicable, the Bonferroni method was used for multiple testing correction.

## 3 Results

### 3.1 The T2-high immune environment in eCRSwNP is comprised of CD4^+^ Th2 cells, ILC2s, and alternatively-activated macrophages

**Figure 1:**
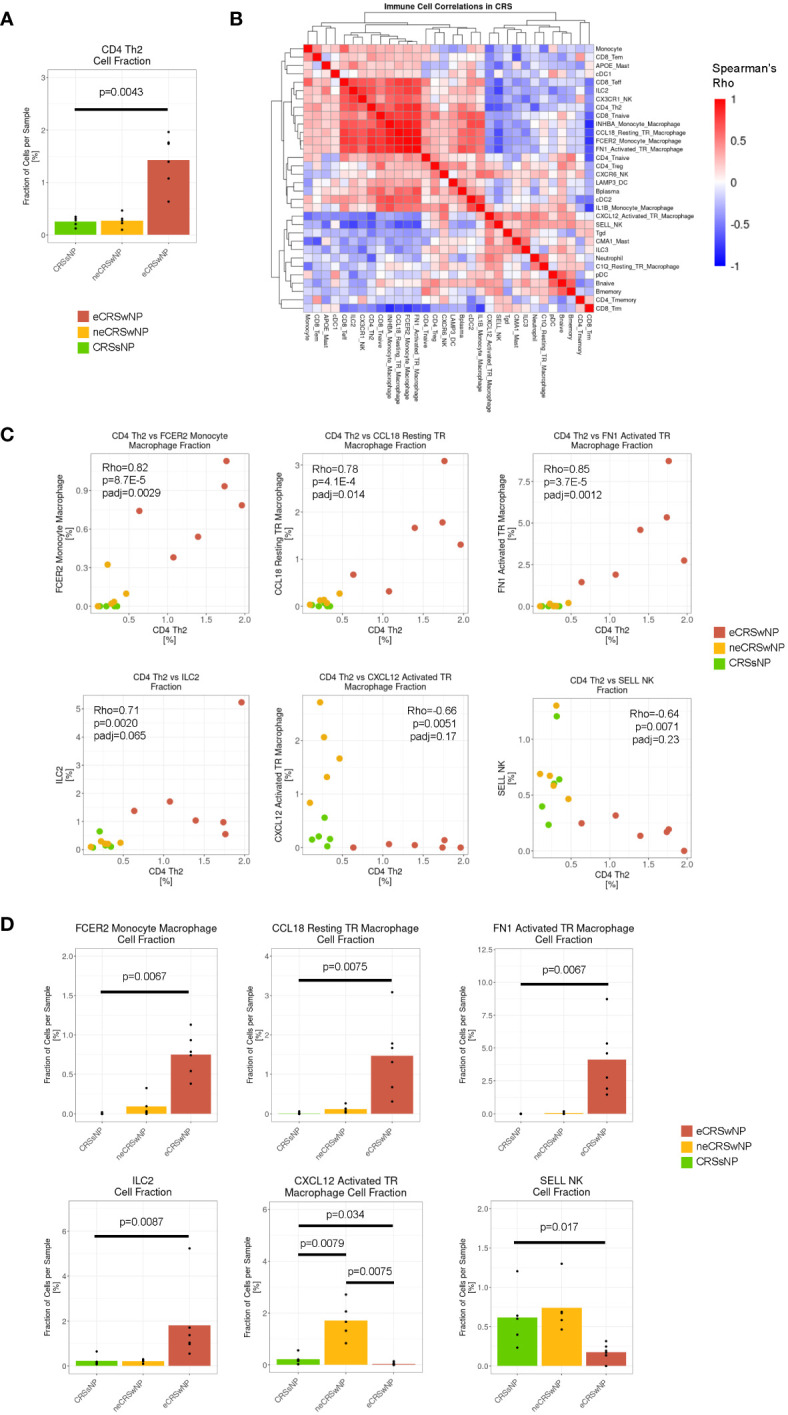
The T2-high immune environment in eCRSwNP is comprised of CD4^+^ Th2 cells, ILC2s, and alternatively-activated macrophages. **(A)** Fraction of CD4 Th2 cells detected per sample (out of total cells per sample) by CRS condition (CRSsNP, neCRSwNP, eCRSwNP). The non-parametric Wilcoxon test was used for statistical testing between CRSsNP and eCRSwNP. **(B)** Correlation matrix of fraction of immune, epithelial, and stromal cell types detected in CRS samples (CRSsNP, neCRSwNP, and eCRSwNP). Color corresponds to Spearman’s *rho*. Rows and columns are hierarchically clustered using the “average” method from the hclust package. **(C)** Scatter plots of CD4 Th2 cell fraction vs selected immune cell populations in CRS samples [FCER2 monocyte-derived macrophages, CCL18 resting tissue-resident macrophages, FN1 activated tissue-resident macrophages, ILC2s, CXCL12 activated tissue-resident macrophages, and SELL NK cells were selected based on top positive and negative correlations from **(B)**]. Each dot indicates 1 sample. *Rho* indicates Spearman’s ρ, p indicates p-value, and padj indicates Bonferroni-corrected p-value. **(D)** Fraction of selected immune cell populations detected per sample (out of total cells per sample) by CRS condition (CRSsNP, neCRSwNP, eCRSwNP). The non-parametric Wilcoxon test was used for statistical testing between CRSsNP and eCRSwNP.

To first survey the immune and structural cell environment in diverse subtypes of CRS, we re-analyzed the recently reported dataset by Wang et al. After integrating with Harmony across disease and sex, we re-clustered immune and structural subsets to obtain 47 cell populations across CRSsNP. As previously reported (13), the population of CD4^+^ Th2 cells were expanded in eCRSwNP samples, as compared to CRSwNP (**Figure 1A**). To define the T2-high immune environment in more detail, we performed a correlation analysis of immune cell frequency across the 16 CRS samples (**Figure 1B**). We observed that CD4^+^ Th2 cells were tightly correlated with 3 populations of macrophages (FCER2 monocyte-derived macrophages, CCL18 resting tissue-resident macrophages, and FN1 activated tissue-resident macrophages) and, as expected, there was also a trend towards correlation with ILC2 cells (**Figure 1C**). Beyond expressing *ALOX15*, as described by Wang et al. (13), these 3 macrophage populations were marked by high levels of *LIPA* and *FN1* (**Supplementary Figure 1B**), features of alternative activation (24–27). Furthermore, there was a trend toward negative correlation of CD4^+^ Th2 cells with CXCL12 activated tissue-resident macrophages and SELL NK cells (**Figure 1C**).

As expected, the 3 populations of alternatively-activated macrophages (FCER2 monocyte-derived macrophages, CCL18 resting tissue-resident macrophages, and FN1 activated tissue-resident macrophages) and ILC2 cells were expanded in eCRSwNP as compared to CRSsNP (**Figure 1D**), while CXCL12 activated tissue-resident macrophages and SELL NK cells were depleted in eCRSwNP. Interestingly, CXCL12 activated tissue-resident macrophages appeared to be exclusive to neCRSwNP (**Figure 1D**), and correlated with Tgd, ILC3, and CMA1 mast cells (**Figure 1B**) which raises the possibility that neCRSwNP and eCRSwNP represent distinct disease endotypes rather than a spectrum of a single entity. In summary, we demonstrate that in CRS the T2-high immune environment involves a coordinated increase in CD4 Th2 cells, ILC2s, and several macrophages with features of alternative activation.

### 3.2 Enhanced glycolytic cell states are detected in epithelial, stromal, and immune cells in eCRSwNP

Our group and others have previously identified enhanced glycolysis in nasal epithelial cells (EpCs) in T2-high eCRSwNP (15, 28). In that study, we performed unsupervised gene set enrichment analysis (GSEA) to demonstrate increased expression of the Hallmark glycolysis gene set in CRSwNP basal EpCs compared to CRSsNP basal EpCs, and we determined that expression of the Hallmark glycolysis gene set was tightly correlated with mTORC1 signaling in CRSwNP basal EpCs and with CD4^+^ Th2 cell infiltration in CRSwNP (15). To extend our metabolic profiling to additional pathways, cells, and disease subsets here, we assessed expression of the Mootha metabolic gene sets (17, 29).

We first validated that the Mootha glycolysis module score in CRS basal EpCs was tightly correlated with the Hallmark glycolysis score (**Supplementary Figure 2A**), mTORC1 signaling (**Supplementary Figure 2B**), and CD4 Th2 cell infiltration (**Supplementary Figure 2C**). We then proceeded with using the Mootha metabolic gene sets to score metabolic pathways - including glycolysis, fatty acid cycle, the citric acid cycle (tricarboxylic acid cycle), and oxidative phosphorylation (OxPhos) - in basal EpCs from the Wang dataset (**Supplementary Figures 2D-G**).

**Figure 2:**
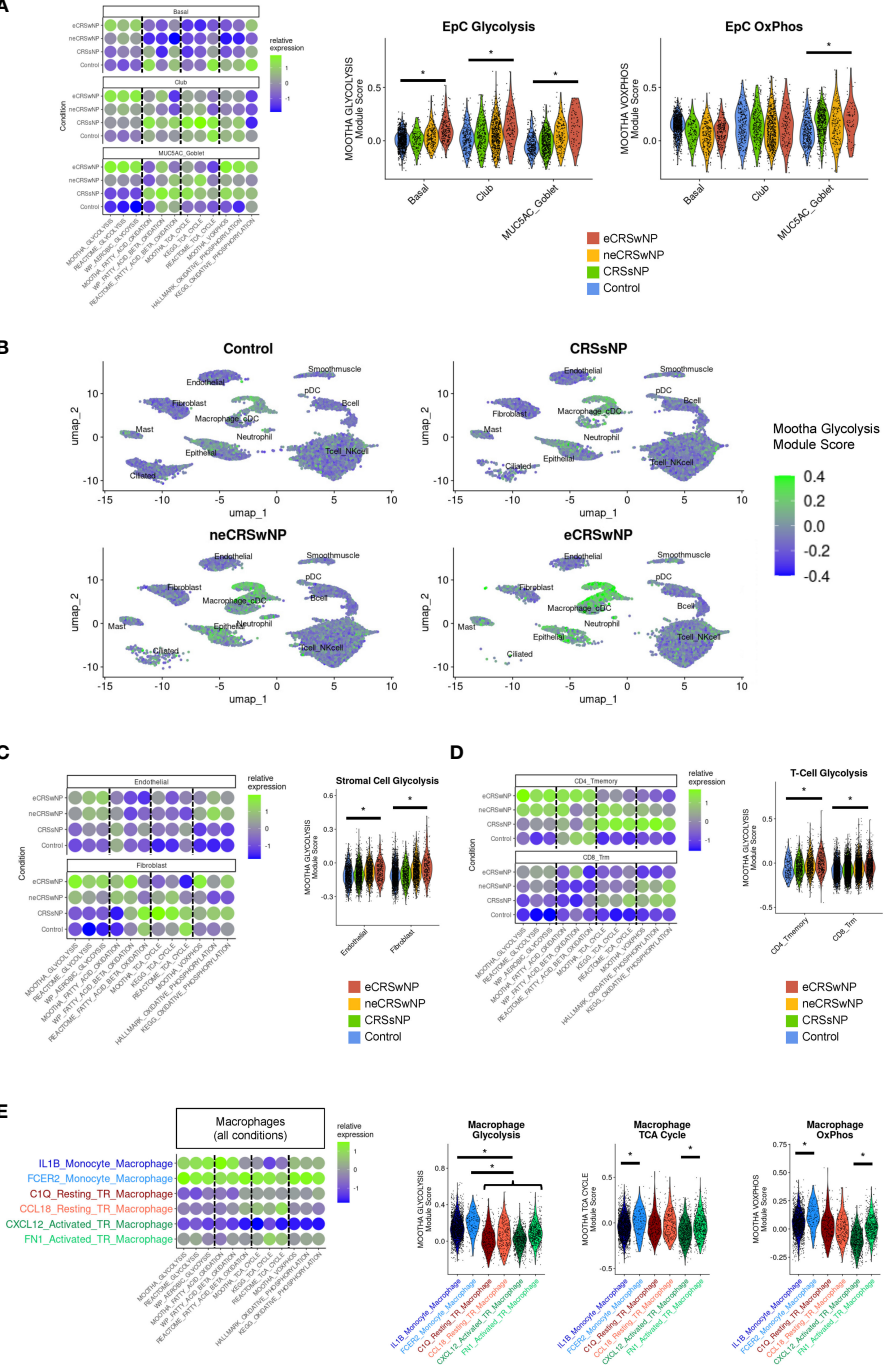
Enhanced glycolytic cell states are detected in epithelial, stromal, and immune cells in eCRSwNP. **(A)** (Left) Dot plot of Mootha metabolic gene set module scores (and similar metabolic gene set module scores) in EpC cell subsets by condition. (Right) Violin plots of Mootha glycolysis and Mootha VOXPHOS module scores in EpC subsets by condition. Wilcoxon test was used for statistical testing between control and eCRSwNP, with each cell treated individually. Asterisk (*) indicates p<0.05. **(B)** Feature plots of Mootha glycolysis module score across the Wang scRNA-seq dataset by condition. **(C)** (Left) Dot plot of Mootha metabolic gene set module scores (and similar metabolic gene set module scores) in stromal cell subsets by condition. (Right) Violin plots of Mootha glycolysis module scores in stromal cell subsets by condition. Wilcoxon test was used for statistical testing between control and eCRSwNP, with each cell treated individually. Asterisk (*) indicates p<0.05. **(D)** (Left) Dot plot of Mootha metabolic gene set module scores (and similar metabolic gene set module scores) in CD4 Tmemory and CD8 Trm cells by condition. (Right) Violin plots of Mootha glycolysis module scores in CD4 Tmemory and CD8 Trm cells by condition. Wilcoxon test was used for statistical testing between control and eCRSwNP, with each cell treated individually. Asterisk (*) indicates p<0.05. **(E)** (Left) Dot plot of Mootha metabolic gene sets module scores (and similar metabolic gene set module scores) in various monocyte-derived and tissue-resident macrophage populations (aggregated across all conditions). (Right) Violin plots of Mootha glycolysis, Mootha TCA cycle, and Mootha VOXPHOS module scores in major macrophage populations aggregated across all conditions. Wilcoxon test was used for statistical testing, with each cell treated individually. Asterisk (*) indicates p<0.05.

Consistent with our prior analysis of EpCs in the Kotas scRNA-seq dataset (30), we observed enhancement of glycolysis in basal and secretory EpCs in eCRSwNP compared to healthy control (**Figure 2A**). In contrast to glycolysis, we did not identify significant increases or decreases in fatty acid oxidation or TCA cycle module scores among basal, club, or MUC5AC goblet EpCs in eCRSwNP vs control (**Figure 2A**). Of note, MUC5AC goblet cells in eCRSwNP demonstrated higher Mootha VOXPHOS module scores than control MUC5AC goblet cells, although there was no comparable upregulation of OxPhos in basal EpCs or club EpCs (**Figure 2A**).

Broad inspection across the Wang dataset revealed that robust expression of glycolytic genes was not limited to epithelial cell clusters (**Figure 2B**). Among stromal cells, we identified enhanced glycolysis in eCRSwNP vs healthy control in endothelial cells and fibroblasts (**Figure 2C**). Among immune cells, we identified enhanced glycolysis in eCRSwNP vs healthy control in CD4^+^ T memory and CD8^+^ T resident memory (CD8^+^ Trm) cells (**Figure 2D**). We also detected increased glycolysis scores in CD4^+^ Th2 cells from eCRSwNP, as compared to CD4^+^ Th2 cells from either healthy control subjects or from subjects with neCRSwNP (**Supplementary Figure 3A**), although this comparison was limited by the scarcity of CD4 ^+^ Th2 in control samples. In contrast to CD8^+^ Trm cells, we did not detect enhanced glycolysis in CD8^+^ Teff or CD8^+^ Tem cells in eCRSwNP vs healthy control (**Supplementary Figure 3B**). However, because T effector cells have been reported to strongly utilize glycolysis (31), we examined glycolysis in T-cell subsets across all samples and observed lower glycolysis scores in naïve CD4^+^ and CD8^+^ T-cells than in the memory and effector T-cell subsets (**Supplementary Figures 3C, D**). While we could not compare metabolism in macrophage populations within each condition (e.g. eCRSwNP vs healthy control) due to limited numbers of alternatively-activated macrophages in controls, we observed across all samples that monocyte-derived macrophages exhibited the highest level of glycolytic gene expression (**Figure 2E**).

Upregulation of TCA cycle and OxPhos genes is reported to be a key feature of macrophage alternative activation (24). Accordingly, we detected enhanced TCA cycle and OxPhos gene expression in monocyte-derived macrophages with features of alternative activation (FCER2 monocyte-derived macrophages), as compared to IL1B monocyte-derived macrophages. Similarly, we detected enhanced TCA cycle and OxPhos gene expression in activated tissue-resident macrophages with features of alternative activation (FN1 activated macrophages), as compared to CXCL12 activated macrophages. Notably, we did not detect upregulation of either TCA gene expression or OxPhos gene expression in resting tissue-resident macrophages with features of alternative activation (CCL18 resting tissue-resident macrophages), as compared to C1Q resting tissue-resident macrophages, suggesting that this metabolic reprogramming accompanies cell activation (**Figure 2E**).

### 3.3 Correlation analysis suggests that enhanced glycolysis in CRS may support tissue remodeling by epithelial, stromal, and immune cells

**Figure 3:**
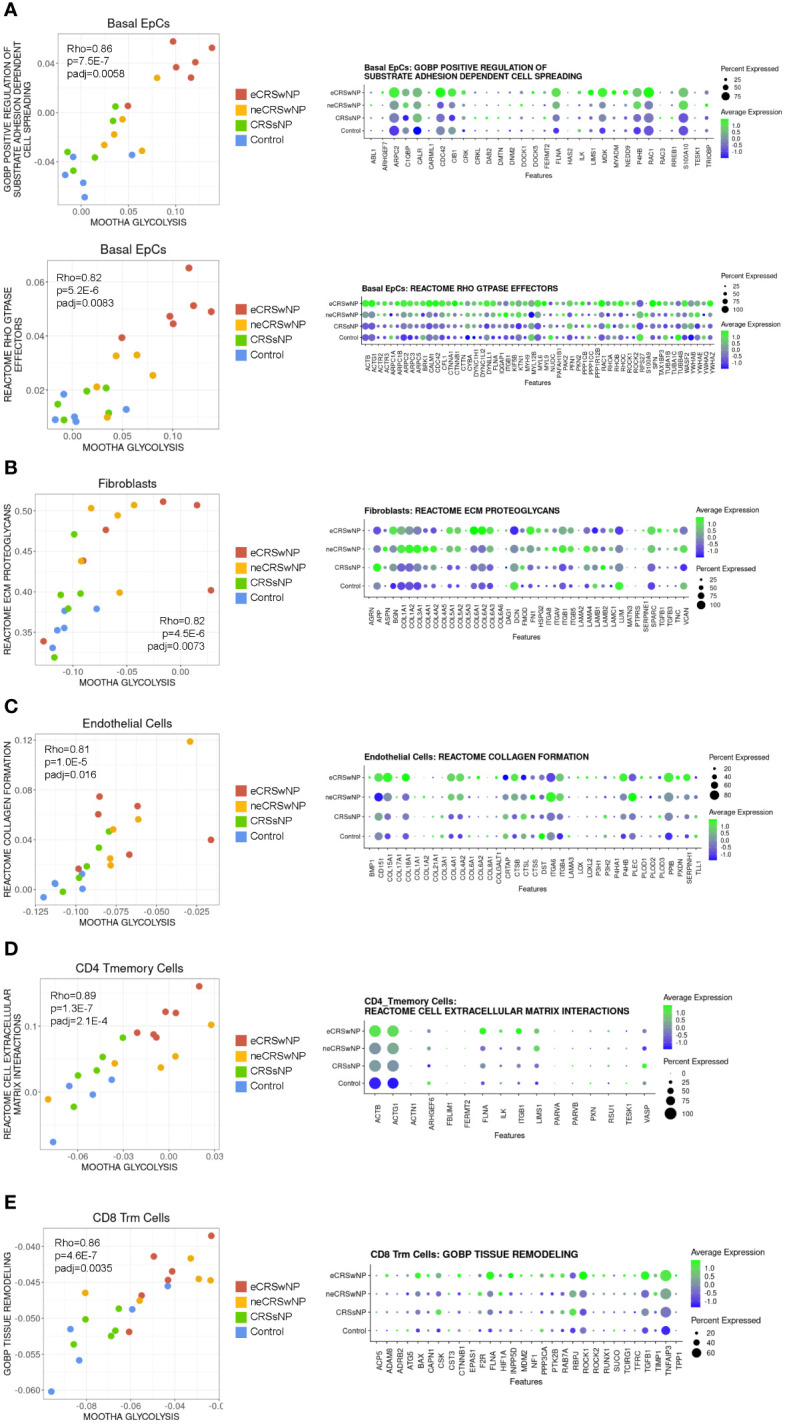
Correlation analysis suggests that enhanced glycolysis in CRS may support tissue remodeling by epithelial, stromal, and immune cells. **(A)** (Left) Scatter plot of Mootha glycolysis module score versus GOBP positive regulation of substrate adhesion dependent cell spreading module score and versus Reactome Rho GTPase effectors module score in basal EpCs across all samples. Each dot indicates 1 sample. *Rho* indicates Spearman’s ρ, p indicates p-value, and padj indicates Bonferroni-corrected p-value. (Right) Dot plot of detected genes in the GOBP positive regulation of substrate adhesion dependent cell spreading gene set and Reactome Rho GTPase effectors gene set in basal EpCs by condition. **(B)** (Left) Scatter plot of Mootha glycolysis module score versus Reactome ECM proteoglycans module score in fibroblasts across all samples. Each dot indicates 1 sample. *Rho* indicates Spearman’s ρ, p indicates p-value, and padj indicates Bonferroni-corrected p-value. (Right) Dot plot of detected genes in the Reactome ECM proteoglycans gene set in fibroblasts by condition. **(C)** (Left) Scatter plot of Mootha glycolysis module score versus Reactome collagen formation module score in endothelial cells across all samples. Each dot indicates 1 sample. *Rho* indicates Spearman’s ρ, p indicates p-value, and padj indicates Bonferroni-corrected p-value. (Right) Dot plot of detected genes in the Reactome collagen formation gene set in endothelial cells by condition. **(D)** (Left) Scatter plot of Mootha glycolysis module score versus Reactome cell extracellular matrix interactions module score in CD4 Tmemory cells across all samples. Each dot indicates 1 sample. *Rho* indicates Spearman’s ρ, p indicates p-value, and padj indicates Bonferroni-corrected p-value. (Right) Dot plot of detected genes in the Reactome cell extracellular matrix interactions gene set in CD4 Tmemory cells by condition. **(E)** (Left) Scatter plot of Mootha glycolysis module score versus GOBP tissue remodeling module score in CD8 Trm cells across all samples. Each dot indicates 1 sample. *Rho* indicates Spearman’s ρ, p indicates p-value, and padj indicates Bonferroni-corrected p-value. (Right) Dot plot of detected genes in the GOBP tissue remodeling gene set in CD8 Trm cells by condition.

To identify cellular functions associated with glycolytic reprogramming in CRS, we performed correlation analyses of the Mootha glycolysis module scores with gene sets in the Gene Ontology Biological Processes database (7655 gene sets) and Reactome database (1615 gene sets), followed by strict Bonferroni multiple testing correction (see **Supplementary Table 1** for all significant results). We previously demonstrated using bulk RNA-seq that sinonasal basal EpC glycolysis is correlated with genes involved in wound healing (15) and, consistent with this, basal EpC glycolysis in the Wang dataset correlated with GOBP and Reactome gene sets related to cell motility (**Figure 3A**). These included genes encoding actin-related protein 2/3 complex (*ARPC2*) (32), the Rho GTPase Cdc42 (*CDC42*) (33), filamin A (*FNLA*) (34), midkine (*MDK*) (35), the scaffold protein NEDD9 (*NEDD9*) (36), and other genes previously reported to control epithelial cell migration, metastasis, and invasion. Basal EpC glycolysis was also highly associated with expression of a broader family of Rho GTPase effectors which were robustly expressed in eCRSwNP.

Among stromal cells, fibroblast glycolysis was positively correlated with the Reactome ECM proteoglycans gene set which includes many collagen genes (**Figure 3B**). Similarly, endothelial cell glycolysis was correlated with the Reactome collagen formation gene set (**Figure 3C**). Interestingly, within T-cells, CD4^+^ T memory cell glycolysis was positively correlated with the Reactome cell extracellular matrix interactions gene set (this gene set includes *ITGB1*) (**Figure 3D**), and CD8^+^ Trm cell glycolysis was positively correlated with the GOBP tissue remodeling gene set (**Figure 3E**). Thus, across stromal and immune populations, enhanced glycolysis is associated with tissue remodeling. Given this picture of tissue remodeling and the basal cell glycolytic association with cell motility, glycolysis in eCRSwNP appears to be associated with a coordinated program of epithelial mesenchymal transition (EMT).

### 3.4 Increased epithelial-stromal and epithelial-immune interactions in eCRSwNP

**Figure 4:**
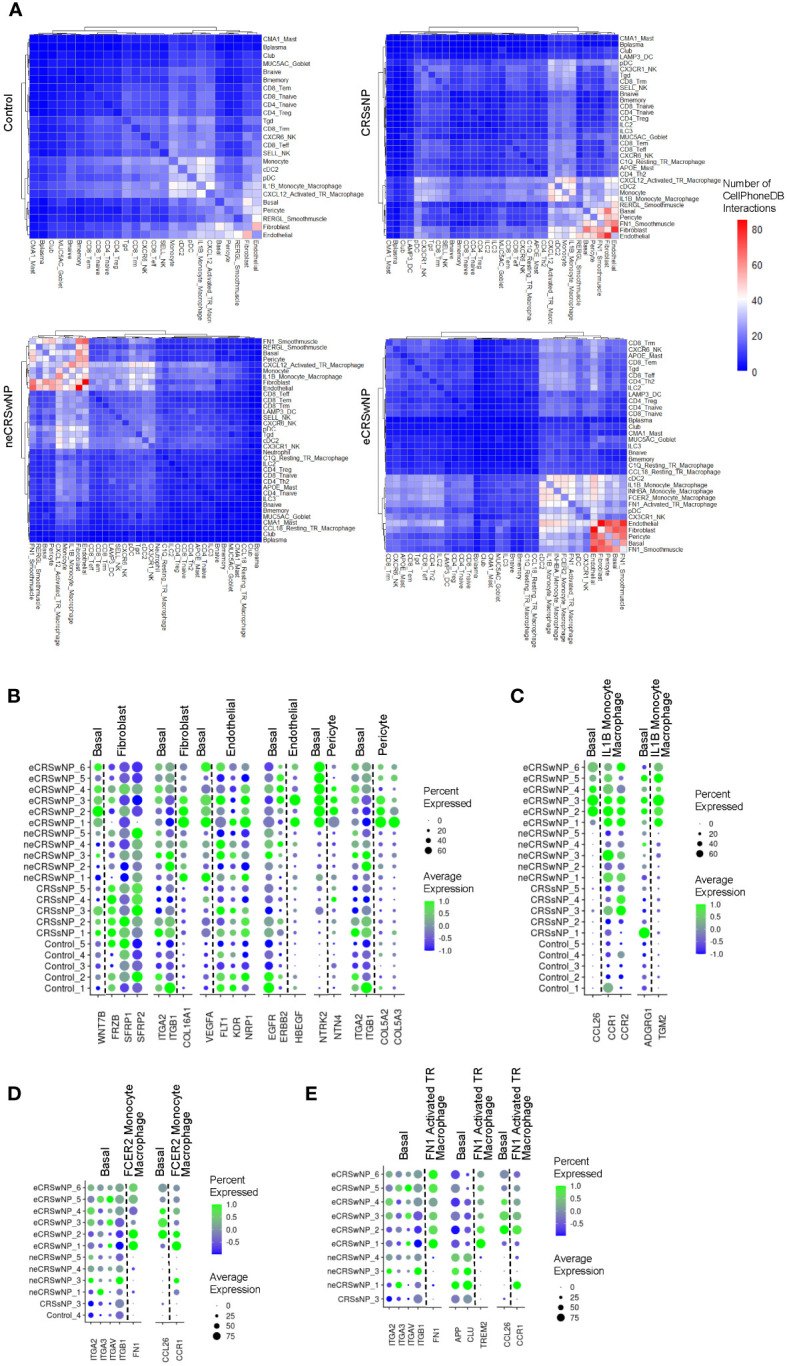
Increased epithelial-stromal and epithelial-immune interactions in eCRSwNP. **(A)** Similarity matrix of CellPhoneDB interactions in detected epithelial, immune, and stromal cell types by condition. Cell types were considered detected within a condition if present in all samples from that condition. Color indicates the average number of interactions nominated by CellPhoneDB across samples for each condition. Rows and columns are hierarchically clustered using the “ward.D2” method from the hclust package. **(B)** Dot plots of genes in selected ligand-receptor interactions between basal EpCs and stromal cells in eCRSwNP but not control (interactions that were significant in at least 5 of 6 eCRSwNP samples and not detected in any control samples). **(C)** Dot plots of genes in selected ligand-receptor interactions between basal EpCs and IL1B monocyte-derived macrophages in eCRSwNP (interactions that were significant in at least 5 of 6 eCRSwNP samples and not detected in any control samples). **(D)** Dot plots of genes in selected ligand-receptor interactions between basal EpCs and FCER2 monocyte-derived macrophages in eCRSwNP (interactions that were significant in at least 5 of 6 eCRSwNP samples and not detected in any non-eCRSwNP samples). **(E)** Dot plots of genes in selected ligand-receptor interactions between basal EpCs and FN1 activated tissue-resident macrophages in eCRSwNP (interactions that were significant in at least 5 of 6 eCRSwNP samples and not detected in any non-eCRSwNP samples).

We next used CellPhoneDB 2.0 to identify possible cell-to-cell interactions across the 21 samples in the Wang dataset. We discovered an increased number of cell-cell interactions in eCRSwNP between basal EpCs and stromal cells as well as between basal EpCs and immune cells (especially macrophages), as compared to healthy controls (**Figure 4A**). Interestingly, within the alternatively-activated macrophage populations, FCER2 monocyte-derived macrophages and FN1 activated tissue-resident macrophages demonstrated a high number of interactions with stromal cells and basal EpCs in eCRSwNP while the CCL18 resting tissue-resident macrophages did not. To identify cell-cell interactions that were unique to eCRSwNP, we focused on cell-cell interactions that were present in at least 5 of 6 eCRSwNP samples and were not present in any of the 5 healthy control samples.

This strategy identified several interactions between basal EpCs and stromal cells in eCRSwNP that were not present in healthy controls (**Figure 4B**), including interactions of *WNT7B* produced by basal EpCs with various fibroblast proteins; interactions of fibroblast *COL16A1* with basal EpC integrins; interactions of basal EpC *VEGFA* with endothelial cell receptors; interactions of endothelial cell *HBEGF* with basal EpC receptors; interactions of pericyte *NTN4* with basal EpC *NTRK2*; and interactions of pericyte *COL5A2* and *COL5A3* with basal EpC integrins. Furthermore, interactions between basal EpCs and IL1B monocyte-derived macrophages that were detected in eCRSwNP but not healthy controls included interaction of basal EpC *CCL26* with macrophage chemokine receptors (*CCR1* and *CCR2*) and interaction of macrophage *TGM2* with basal EpC *ADGRG1* (**Figure 4C**).

We also investigated interactions of FCER monocyte-derived macrophages and FN1 activated tissue-resident macrophages with basal EpCs, as these populations were expanded in eCRSwNP (**Figure 1D**) and exhibited a high degree of connectivity with epithelial and stromal cells in eCRSwNP (**Figure 4A**). We could not filter out interactions detected in all 5 control samples because these 2 alternatively-activated macrophage populations were largely absent in healthy controls, therefore we instead identified interactions which were present in 5 of 6 eCRSwNP samples and were not present in any non-eCRSwNP samples. This approach identified interactions of FCER2 monocyte-derived macrophage *FN1* with basal EpC integrins and interaction of basal EpC *CCL26* with FCER2 monocyte-derived macrophage *CCR1* (**Figure 4D**). Similarly, we detected interactions of FN1 activated tissue-resident macrophage *FN1* with basal EpC integrins, basal EpC *CCL26* with FN1 activated tissue-resident macrophage *CCR1*, and also an integration of FN1 activated tissue-resident macrophage *TREM2* with basal EpC receptors (**Figure 4E**).

Finally, we performed a similar analysis to identify endothelial-immune interactions, as endothelial cells exhibited a high degree of connectivity with several macrophage populations in eCRSwNP (**Figure 4A**). Similar to what we observed with basal EpCs, we also identified interactions of macrophage *TGM2* with endothelial cell *ADGRG1* (**Supplementary Figure 4A**), interactions of FCER2 monocyte-derived macrophage *FN1* with endothelial cell integrins (**Supplementary Figure 4B**), and interactions of FN1 activated tissue resident macrophage *TREM2* with endothelial cell receptors (**Supplementary Figure 4C**).

## 4 Discussion

### 4.1 Altered in cell composition in eCRSwNP

In this report, we identify 3 populations of alternatively-activated macrophages (FCER2 monocyte-derived macrophages, CCL18 resting tissue-resident macrophages, and FN1 activated tissue-resident macrophages) as being a central component of the T2-high immune environment in eCRSwNP, in addition to CD4 Th2 cells and ILC2s. Wang et al. previously observed that these macrophage populations were notable for high expression of *ALOX15*, and that *in vitro ALOX15* inhibition in macrophages resulted decreased inflammatory cytokine expression as well as impaired recruit of CD4 T-cells by macrophage conditioned media (13), highlighting a potential pro-inflammatory circuit between lymphocytes and macrophages that drives T2 inflammation. *In vitro* studies of epithelial and stromal cells frequently simulate T2-high immune environments with addition of the classical Th2 cytokines IL-4, IL-5, and IL-13, but fail to recapitulate many epithelial genes that are differentially expressed in T2-high asthma or nasal polyps *in vivo*. This study highlights that macrophage soluble products may account for some of these discrepancies. Notably, recent studies in both humans and mice have identified the importance of macrophage-epithelial interactions in wound repair and asthma (37, 38).

### 4.2 Altered in immunometabolism in eCRSwNP

We previously identified that enhanced glycolysis in CRSwNP basal EpCs correlates with the expression of EpC wound healing genes (15). In this study, we sought to investigate if enhanced glycolysis was unique to EpCs within CRSwNP tissue. We observed that enhanced glycolysis is a feature of various stromal cell populations in CRSwNP, in addition to EpCs. Furthermore, glycolytic gene expression was positively correlated with ECM remodeling gene sets in fibroblasts and endothelial cells, specifically with gene sets involving collagen synthesis (**Figures 3B, C**). This is consistent with prior reports which have nominated inhibition of fibroblast glycolysis as a therapeutic target for treatment of fibrotic diseases (39) and studies demonstrating that inhibition of endothelial cell glycolysis can decrease endothelial proliferation and recruitment of inflammatory cells (40, 41). Interestingly, the positive correlation between glycolysis and the Reactome collagen formation gene set in endothelial cells appeared to be driven by *COL15A1* and *COL18A1* (**Figure 3C**), both collagens which can be proteolytically cleaved to release signaling domains that regulate angiogenesis (42, 43). Overall, these concordant alterations in immunometabolism suggest the possibility of a common signal driving enhanced glycolysis in structural cells in CRSwNP (for example, tissue hypoxia or locally-secreted cues such as growth factors) and we propose that the use of spatial transcriptomics may elucidate this in future studies.

Within immune cells, we also observed enhanced glycolytic cell states in memory T-cell populations (CD8 Trm and CD4 Tmemory) in eCRSwNP compared to healthy controls. Although we observed significant positive correlations of glycolysis in these cell types with tissue remodeling gene sets (**Figure 3**), there are likely additional functions of this metabolic reprograming beyond cell-matrix interactions. For example, induction of glycolysis in memory CD8 T-cells has been reported to impair their long-term survival and enhance the differentiation of memory cells into effector CD8 T-cell (44). Consistent with this, we observed depletion of CD8 Trm cells in eCRSwNP and accumulation of CD8 Teff cells which paralleled the alterations in CD8 Trm glycolytic metabolism (**Supplementary Figure 1C**).

Within macrophage populations, we observed enhanced expression of TCA cycle and OxPhos genes in alternatively-activated populations of monocyte-derived macrophages and activated tissue-resident macrophages (**Figure 2C**), consistent with prior reports (24). We did not observe enhanced TCA cycle or OxPhos in alternatively-activated resting tissue-resident macrophages (CCL18 resting tissue-resident macrophages), perhaps consistent with their resting behavior. Wang et al. suggest that *ALOX15^+^* macrophages may play a key role in tissue remodeling in CRSwNP pathogenesis (13), and in support of this hypothesis we detected an increased number of cell-cell interactions between several macrophage populations with epithelial and stromal cells in eCRSwNP (**Figure 4A**). Interestingly, the distribution of cell-cell interactions in alternatively-activated macrophages paralleled glycolytic reprograming in that FCER2 monocyte-derived macrophages and FN1 activated tissue-resident macrophages exhibited a high number of interactions with other cells, while CCL18 resting tissue-resident macrophages did not (even though the CCL18 resting tissue-resident macrophage was expanded in eCRSwNP as shown in **Figure 1D**). Overall, this supports the conclusion that glycolytic rewiring may underly cell-cell and cell-matrix interactions in CRSwNP.

### 4.3 Altered epithelial-stromal interactions in eCRSwNP

Our analysis using CellPhoneDB identified increased cell-cell interactions in eCRSwNP (**Figure 4A**), most prominently between basal EpCs and stromal cells and between basal EpCs and immune cells. Surprisingly, we did not detect a high number of interactions involving CD4 Th2 cells or non-basal epithelial cells (club EpCs and MUC5AC goblet cells) (**Figure 4A**), which underscores the central role of the basal epithelium in CRSwNP and is consistent with prior studies (10, 11).

As expected, several interactions between basal EpCs and stromal cells in eCRSwNP involved secreted ECM proteins. Basal EpCs were detected to interact with fibroblast *COL16A1*, a gene which has been implicated in GWAS of CRSwNP and asthma (45, 46). Similarly, in eCRSwNP we detected an interaction of basal EpCs with pericyte *COL5A2* and *COL5A3*, both of which have been reported to be differentially methylated in allergic asthma (47).

Other basal EpC-stromal cell interactions detected in eCRSwNP involved more conventional ligand-receptor interactions. For example, we detected a possible interaction of basal EpC *VEGF* with receptors on endothelial cells (**Figure 4B**). This is consistent with several reports identifying increased *VEGF* in nasal polyp tissue (48, 49), and in the lower airway *VEGFA* from EpCs has been implicated in signaling to endothelial cells to promote angiogenesis in obstructive lung disease (50, 51). We also detected a possible interaction of endothelial cell *HB-EGF* (heparin-binding EGF-like growth factor) with basal EpC receptors (**Figure 4B**), which may be involved in regulating EpC tissue remodeling in CRSwNP. *HB-EGF* signaling has been shown to induce expression of matrix metalloproteinases (MMPs) in airway EpCs (52); while *HB-EGF* has not been found to elevated in nasal polyps at the whole tissue level (52), our findings implicate endothelial cells as a potentially relevant source of this growth factor *in vivo*.

CellPhoneDB also detected a possible interaction of basal EpC *WNT7B* with fibroblast receptors, including the secreted receptors *SFRP1* and *SFRP2*, which act to prevent binding of Wnt ligand to canonical frizzled receptors (53). Consistent with a prior report identifying higher expression of *WNT7B* in nasal polyp tissue compared with donor-matched inferior turbinate tissue (53), we observed high expression of *WNT7B* in nasal polyp basal EpCs (**Figure 4B**). In general, Wnt signaling has been reported to promote release of EpC cytokines and loss of adhesion in human nasal EpCs (53). Furthermore, *WNT7B* is a downstream target of the basal stem cell transcription factor p63 in EpCs (54), therefore elevated *WNT7B* signaling may be related to the impaired basal EpC differentiation that has been observed in CRSwNP (10, 11). Interestingly, within the CRS subtypes, there was very high expression of *SFRP1* and *SFRP2* in CRSsNP (**Figure 4B**), which suggests that fibroblasts may regulate EpC Wnt signaling in CRS and highlights the possibility that unopposed EpC Wnt signaling may contribute to nasal polyp formation.

Furthermore, CellPhoneDB implicated a possible interaction between pericyte *NTN4* and basal EpC *NTRK2* in eCRSwNP. *NTRK2* is a canonical T2-inducible gene in airway EpCs (11, 30), although its exact function in the airway epithelium remains unclear. Whereas prior studies have examined the ligand *BDNF* with varying results (55), this analysis implicates that *NTN4*, which is known to be expressed by pericytes (56) and functions to regulate EMT in epithelial malignancies (57), may be a potentially relevant ligand in CRSwNP *in vivo*.

### 4.4 Altered epithelial-immune interactions in eCRSwNP

Between basal EpCs and various macrophage populations, CellPhoneDB analysis identified several possible interactions involving macrophage ligands with known function in immunomodulation and alternative activation. For example, in eCRSwNP we detected an interaction between IL1B monocyte-derived macrophage *TGM2*, which is a marker of alternative interaction (58), and basal EpC *ADGRG1*, a receptor which has been implicated in epithelial-mesenchymal transition in epithelial tumors (59) (**Figure 4C**). Similarly, in eCRSwNP, FN1 activated tissue-resident macrophages were observed to interact with basal EpCs via *TREM2*, which is a marker of tumor-associated macrophages (60, 61). We also detected interactions between *FN1* produced by alternatively-activated macrophages and basal EpCs via epithelial *ITGB1* (integrin beta 1) in eCRSwNP (**Figures 4D, E**). *ITGB1* signaling in basal EpCs has been reported to be critical for EpC stem cell function in skin and mammary tissue (62, 63), therefore macrophage expression of *FN1* may provide a signal that regulates basal EpC stemness and differentiation capacity.

Finally, we also detected possible interactions of basal EpC *CCL26* with *CCR1* on IL1B monocyte-derived macrophages, FCER2 monocyte-derived macrophages, and FN1 activated tissue-resident macrophages in eCRSwNP. *CCL26*, which encodes the well-described eosinophil chemoattractant eotaxin-3, is also a canonical T2 response gene in the airway epithelium (64, 65). Interestingly, we also detected an interaction of basal EpC *CCL26* with a different chemotactic receptor, *CCR2*, on IL1B monocyte-derived macrophages in eCRSwNP, but not in FCER2 monocyte-derived macrophages or FN1 activated tissue-resident macrophages in eCRSwNP. Macrophages expressing *CCR2* have been implicated in asthma pathogenesis due to their expansion after allergen challenge in human asthmatics (37) and have been identified as potential precursors for intra-epithelial macrophages and alternatively-activated macrophages in mouse asthma models (38). Whereas *CCR2* is conventionally thought of as a receptor for the monocyte chemoattractant *CCL2* (which encodes MCP-1), binding of *CCL26* to *CCR2* has been reported to prevent the ability of monocytes to response to monocyte chemokines such as MCP-1 (66); therefore we propose that this *CCL26-CCR2* interaction may underlie the lack of expansion in IL1B monocyte-derived macrophages in eCRSwNP (**Supplementary Figure 1C**) compared to alternatively-activated macrophage populations (**Figure 1D**).

### 4.5 Summary

In conclusion, here we harnessed the diversity of CRS and advances in scRNA-seq to identify metabolic rewiring and identify aberrant cell-cell interactions in eCRSwNP, and we identify that these behaviors appear to support tissue remodeling in CRSwNP.

## Data availability statement

Publicly available datasets were analyzed in this study. This data can be found here: Genome Sequence Archive For Human. Accession Number: HRA000772. https://ngdc.cncb.ac.cn/gsa-human/browse/HRA000772.

## Author contributions

NB: Conceptualization, Data curation, Formal Analysis, Funding acquisition, Project administration, Resources, Supervision, Visualization, Writing – review & editing. GH: Conceptualization, Data curation, Formal Analysis, Investigation, Visualization, Writing – original draft, Writing – review & editing. MM: Conceptualization, Data curation, Formal Analysis, Visualization, Writing – original draft. SD: Formal Analysis, Writing – original draft. DF-S: Formal Analysis, Writing – original draft. MG: Conceptualization, Formal Analysis, Supervision, Validation, Visualization, Writing – review & editing.
