# Supplementary material for: Increased glycolysis and cellular crosstalk in eosinophilic chronic rhinosinusitis with nasal polyps
Source: Front Immunol. 2024 Feb 20;15:1321560. doi: 10.3389/fimmu.2024.1321560 (PMC10912276; doi:10.3389/fimmu.2024.1321560)
Supplement: Supplementary file 1 [file DataSheet_1.pdf]

Supplementary Material

Increased glycolysis and cellular crosstalk in eosinophilic chronic rhinosinusitis with nasal polyps (eCRSwNP)

George X. Huang, MD[1,2]; Michael V. Mandanas, BS[1]; Sarah Djeddi, PhD[3]; Daniela Fernandez, BA[3]; Maria Gutierrez-Arcelus, PhD[3]; Nora A. Barrett, MD[*,1,2]

[1]Division of Allergy and Clinical Immunology, Brigham and Women's Hospital, Boston, MA, United States of America

[2]Department of Medicine, Harvard Medical School, Boston, MA, United States of America

[3]Division of Immunology, Boston Children's Hospital, Boston, MA, United State of America

***Correspondence:**
Nora A. Barrett
Hale Building for Transformative Medicine, Room 05002R
60 Fenwood Road
Boston, MA 02115
nbarrett@bwh.harvard.edu
tel (617) 525-1270

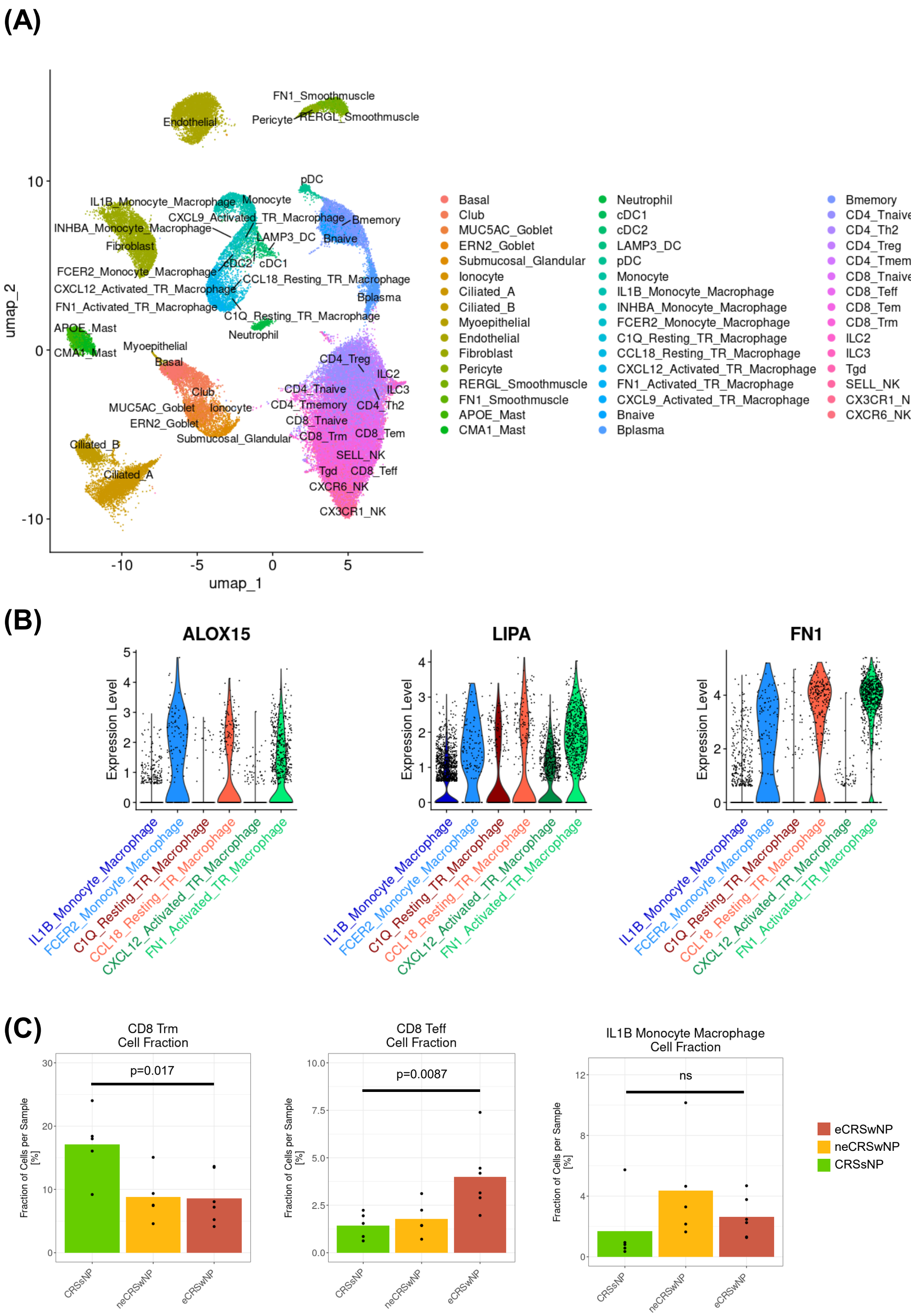

Supplementary Figure 1. (A) UMAP of the Wang scRNA-seq dataset after independent clustering and sub-clustering. Color corresponds to 47 detected cell types. **(B)** Violin plots of *ALOX15, LIPA,* and *FN1* expression in major macrophage populations aggregated across all samples. **(C)** Fraction of CD8 Trm cells, CD8 Teff cells, and IL1B monocyte-derived macrophages detected per sample (out of total cells per sample) by CRS condition (CRSsNP, neCRSwNP, eCRSwNP). The non-parametric Wilcoxon test was used for statistical testing between CRSsNP and eCRSwNP. ns indicates p>0.05.

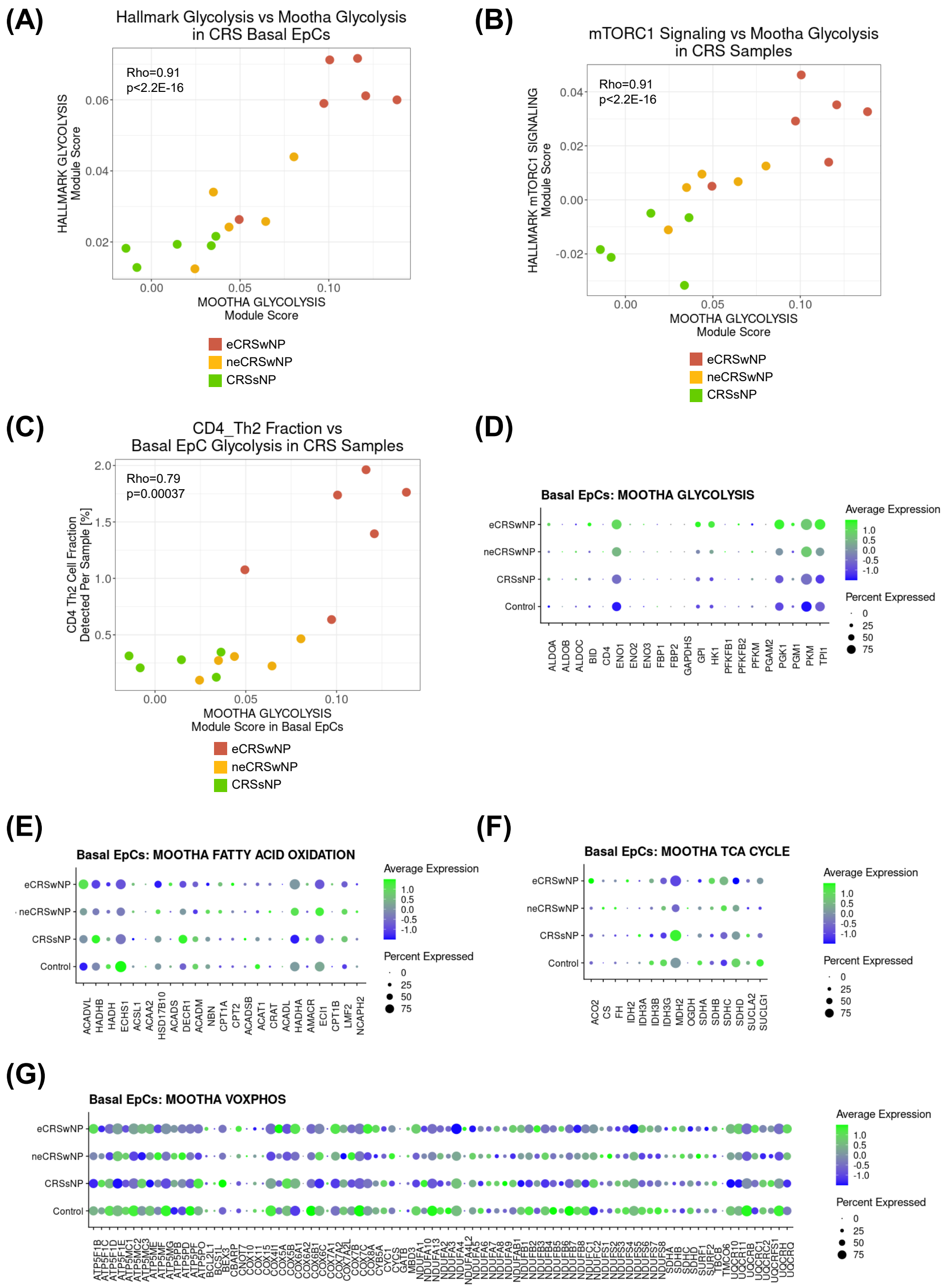

Supplementary Figure 2. (A) Scatter plot of Mootha glycolysis module score vs Hallmark glycolysis module score in basal EpCs across CRS samples. Each dot indicates 1 sample. *Rho* indicates Spearman's ρ and p indicates p-value. **(B)** Scatter plot of Mootha glycolysis module score vs Hallmark mTORC1 signaling module score in basal EpCs across CRS samples. Each dot indicates 1 sample. *Rho* indicates Spearman's ρ and p indicates p-value. **(C)** Scatter plot of Mootha glycolysis module score in basal EpCs vs CD4 Th2 cell fraction across CRS samples. Each dot indicates 1 sample. *Rho* indicates Spearman's ρ and p indicates p-value. **(D)** Dot plot of detected genes in the Mootha glycolysis gene set in basal EpCs by condition. **(E)** Dot plot of detected genes in the Mootha fatty acid oxidation gene set in basal EpCs by condition. **(F)** Dot plot of detected genes in the Mootha TCA cycle gene set in basal EpCs by condition. **(G)** Dot plot of detected genes in the Mootha VOXPHOS gene set in basal EpCs by condition.

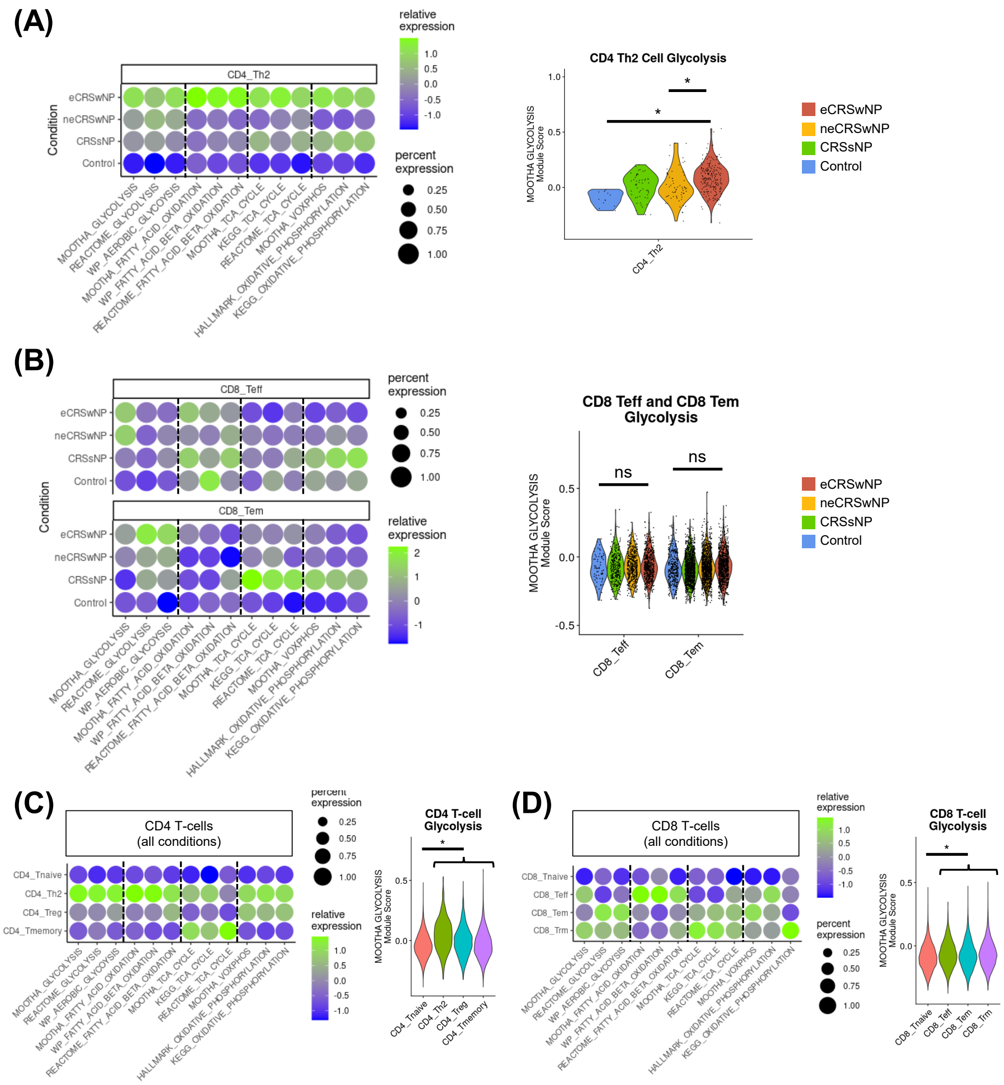

Supplementary Figure 3. (A) Dot plot of Mootha metabolic gene set module scores (and similar metabolic gene set module scores) in CD4 Th2 cells by condition. (Right) Violin plot of Mootha glycolysis module scores in CD4 Th2 cells by condition. The Wilcoxon test was used for statistical testing for eCRSwNP vs control and eCRSwNP vs neCRSwNP, with each cell treated individually. Asterisk (*) indicates $p<0.05$. **(B)** Dot plot of Mootha metabolic gene set module scores (and similar metabolic gene set module scores) in CD8 Teff and CD8 Tem cells by condition. (Right) Violin plot of Mootha glycolysis module scores in CD8 Teff and CD8 Tem cells by condition. The Wilcoxon test was used for statistical testing for eCRSwNP vs control, with each cell treated individually. ns indicates $p>0.05$. **(C)** (Left) Dot plot of Mootha metabolic gene set module scores (and similar metabolic gene set module scores) in CD4 T-cell subsets (aggregated across all conditions). (Right) Violin plot of Mootha glycolysis module scores in CD4 T-cell subsets aggregated across all conditions. Wilcoxon test was used for statistical testing, which each cell treated individually. Asterisk (*) indicates $p<0.05$. **(D)** (Left) Dot plot of Mootha metabolic gene set module scores (and similar metabolic gene set module scores) in CD8 T-cell subsets (aggregated across all conditions). (Right) Violin plot of Mootha glycolysis module scores in CD8 T-cell subsets aggregated across all conditions. Wilcoxon test was used for statistical testing, which each cell treated individually. Asterisk (*) indicates $p<0.05$.

(A)

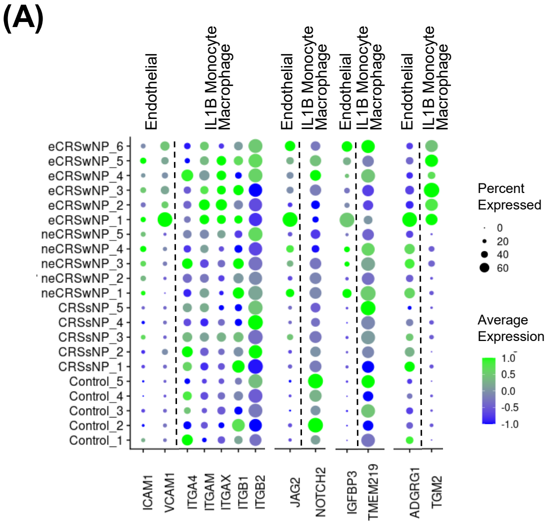

(B)

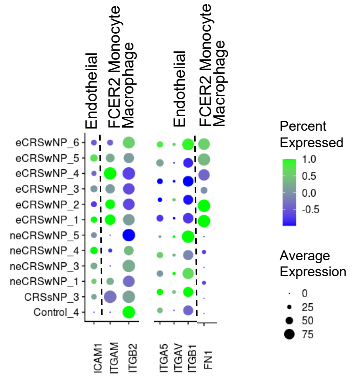

(C)

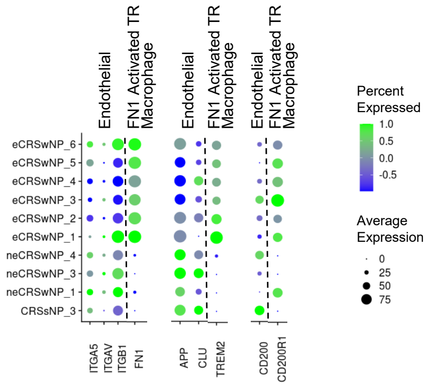

Supplementary Figure 4. (A) Dot plots of genes in selected ligand-receptor interactions between endothelial cells and IL1B monocyte-derived macrophages in eCRSwNP (interactions that were significant in at least 5 of 6 eCRSwNP samples and not detected in any control samples). **(B)** Dot plots of genes in selected ligand-receptor interactions between endothelial cells and FCER2 monocyte-derived macrophages in eCRSwNP (interactions that were significant in at least 5 of 6 eCRSwNP samples and not detected in any non-eCRSwNP samples). **(C)** Dot plots of genes in selected ligand-receptor interactions between endothelial cells and FN1 activated tissue-resident macrophages in eCRSwNP (interactions that were significant in at least 5 of 6 eCRSwNP samples and not detected in any non-eCRSwNP samples).
